# Cellulose Acetate Butyrate Films as a Platform for Energy Upconversion Composites: Design and Properties

**DOI:** 10.1021/acsomega.5c02294

**Published:** 2025-04-24

**Authors:** Matheus V. B. Silva, York E. Serge-Correales, Lucas H. Pereira, Hernane da S. Barud, Sidney J. L. Ribeiro, Harumi Otaguro, Rosana M. N. de Assunção

**Affiliations:** † Institute of Chemistry, Federal University of Uberlândia, Uberlândia, Minas Gerais 38408-100, Brazil; ‡ Institute of Chemistry, State University of São Paulo, Araraquara, São Paulo 14800-060, Brazil; § Biopolymers and Biomaterials Laboratory, University of Araraquara, Araraquara 14801-340, Brazil; ∥ Center for Marine Studies, Federal University of Paraná, Pontal do Paraná, Paraná 83255-976, Brazil; ⊥ Institute of Exact and Natural Sciences of Pontal, Federal University of Uberlândia, Ituiutaba, Minas Gerais 38304-402, Brazil

## Abstract

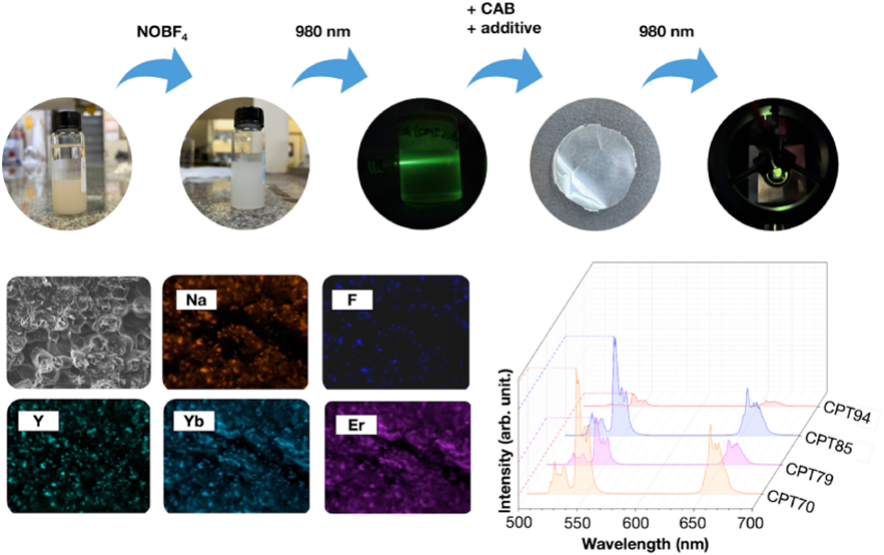

This study presents the development of composite films made from cellulose acetate butyrate (CAB) incorporating NaYF_4_:Yb^3+^,Er^3+^ upconversion particles. The films were synthesized by using a solvent casting method, focusing on optimizing their structural, thermal, and luminescent properties. The upconversion particles were produced via a hydrothermal method and then surface-modified using nitrosonium tetrafluoroborate (NOBF_4_) to enhance dispersion in polar solvents. Including upconversion particles notably increased the luminescence performance of the films, while adding Tween 80 and dioctyl sodium sulfosuccinate (DSS) significantly improved mechanical and optical characteristics. The structural, morphological, and luminescence properties were thoroughly evaluated by using scanning electron microscopy (SEM), X-ray diffraction (XRD), thermogravimetric analysis (TGA), and photoluminescence spectroscopy. Results demonstrated a uniform distribution of particles with well-preserved upconversion properties, revealing that Tween 80 contributed to a higher luminescence intensity than DSS. These findings underscore the potential of CAB-based composites in photonic device applications, offering a promising avenue for advancing energy conversion technologies.

## 1 Introduction

Since the pioneering work of Auzel et al.,^1^ where researchers synthesized NaYF4:Yb^3+^,Er^3+^ particles with upconversion energy properties and incorporated them into glass matrices, interest in developing processes for the production of these particles has grown significantly.^1,2^ The ability of these particles to emit photoluminescence when excited by low-energy light sources, such as near-infrared (NIR) light, allows them to exhibit unique optical properties. This phenomenon occurs due to their ability to convert low-energy photons into high-energy emissions, resulting in distinct spectral characteristics. This capability makes these particles particularly useful in various applications, including materials for 3D displays, photovoltaic devices, bioimaging, security detection, and photonic devices, where the deep penetration and selectivity of NIR light are essential.^2,3^

However, the successful application of upconversion luminescent particles and nanoparticles depends on several fundamental properties, such as average particle size, good dispersion, and high fluorescence intensity. Traditional synthetic methods often result in particles with low hydrophilicity, typically coated with hydrophobic surfactants, such as oleate, to prevent aggregation.^4^ To overcome this limitation, new surface modification methods have been developed, including ligand exchange, ligand oxidation, polymer functionalization, and surface encapsulation.^4^ These strategies allow for the adjustment of the particles’ surface properties, enhancing their functionality and compatibility in diverse environments. For instance, oleic acid (OA) and oleylamine (OM) are frequently used because these hydrophobic ligands can be exchanged with end-functional polymers that strongly bind to the surface of upconversion particles (UCPs).^5^ These end-functional polymers act as capping agents or surfactants, crucial for determining the particles’ morphology, shape, size, and homogeneous distribution. Another approach involves grafting UCPs with polymers of varying molecular weights and types, such as polyvinylpyrrolidone (PVP), poly(acrylic acid) (PAA), polyethylene glycol (PEG), and polyethylenimine (PEI).^5−7^

In this context, the production of composites or nanocomposites has proven to be an effective approach to integrate inorganic materials, such as upconversion nanoparticles (UCNPs), into organic polymer matrices, with the dispersion process playing a key role in ensuring homogeneous distribution and optimal performance. The combination of these components results in a synergistic effect, adding crucial properties such as processability while maintaining or enhancing important physicochemical characteristics, including mechanical, thermal, electrical, and optical properties.^8,9^ Additionally, the selected polymer must interact well with the nanoparticles to ensure photoluminescence, transparency, and the ability to form films or be molded into various shapes.^9^

Numerous studies have investigated the use of polymers such as polystyrene (PS),^3,10^ polyamide (PA),^10,11^ poly(methyl methacrylate) (PMMA),^3,12^ polydimethylsiloxane (PDMS),^10^ and cellulose and its derivatives^9,13−15^ as matrices for polymer nanocomposites incorporating UCNPs. These polymers are selected for their unique properties, which enable the development of multifunctional materials tailored for advanced technological applications. Among these, poly(vinyl alcohol) (PVA)^8^ is widely recognized for its transparency and ease of processing, making it an excellent choice for producing luminescent films used in anticounterfeiting technologies, packaging, and displays. Polyamide/poly(methyl methacrylate) (PA6/PMMA)^11^ combines high thermal and mechanical stability, making it suitable for applications in optical fibers and photonic devices. Cellulose nanocrystals (CNCs)^13^ stand out for their exceptional transparency and biodegradability, positioning them as a promising material for optical sensors and reusable paper-based devices.

Particularly, cellulose acetate (CA)^15^ and cellulose acetate butyrate (CAB)^16^ stand out for their superior film-forming properties, which are essential for creating uniform and transparent films. These polymers also provide excellent moisture protection, making them highly suitable for applications, such as drug delivery capsules and optical thermometry. However, to the best of our knowledge, there are no studies in the literature employing CAB as a matrix for producing composites with NaYF4:Yb^3+^,Er^3+^ microparticles. This study addresses this gap by employing CAB as the polymer matrix to develop a stable composite with energy upconversion properties. Cellulose derivatives, such as CAB, are widely available due to the abundance of cellulose, and their physicochemical properties can be tailored through chemical modifications. CAB stands out for its higher hydrophobicity and excellent film-forming capabilities, making it particularly effective for dispersing NaYF4:Yb^3+^,Er^3+^ microparticles and producing composites with a transparent polymer matrix. These properties make CAB a promising candidate for applications requiring moisture protection, such as humidity sensors and optical devices. However, films produced with CAB tend to be more rigid and brittle compared with those made with cellulose acetate (CA). This rigidity can be mitigated by incorporating plasticizers into the mixture.

In this work, we aimed to develop CAB-based upconversion composite films and optimize their structural, thermal, and luminescent properties. Composite films containing NaYF4:Yb^3+^,Er^3+^ luminophores were successfully produced by using cellulose acetate butyrate (CAB) as the polymeric matrix. To further enhance the film properties, two additives, Tween 80 and dioctyl sodium sulfosuccinate (DSS), were incorporated. These additives not only improved the flexibility and durability of the films but also promoted the homogeneous dispersion of the microparticles within the matrix. The unique attributes of CAB, combined with the upconversion properties of NaYF4:Yb^3+^,Er^3+^ microparticles, make this composite a promising material for advanced applications, including optical thermometry, humidity sensing, and moisture-resistant coatings.

## 2 Results and Discussion

### 2.1 Morphology and Structure of Particles

**Figure 1:**
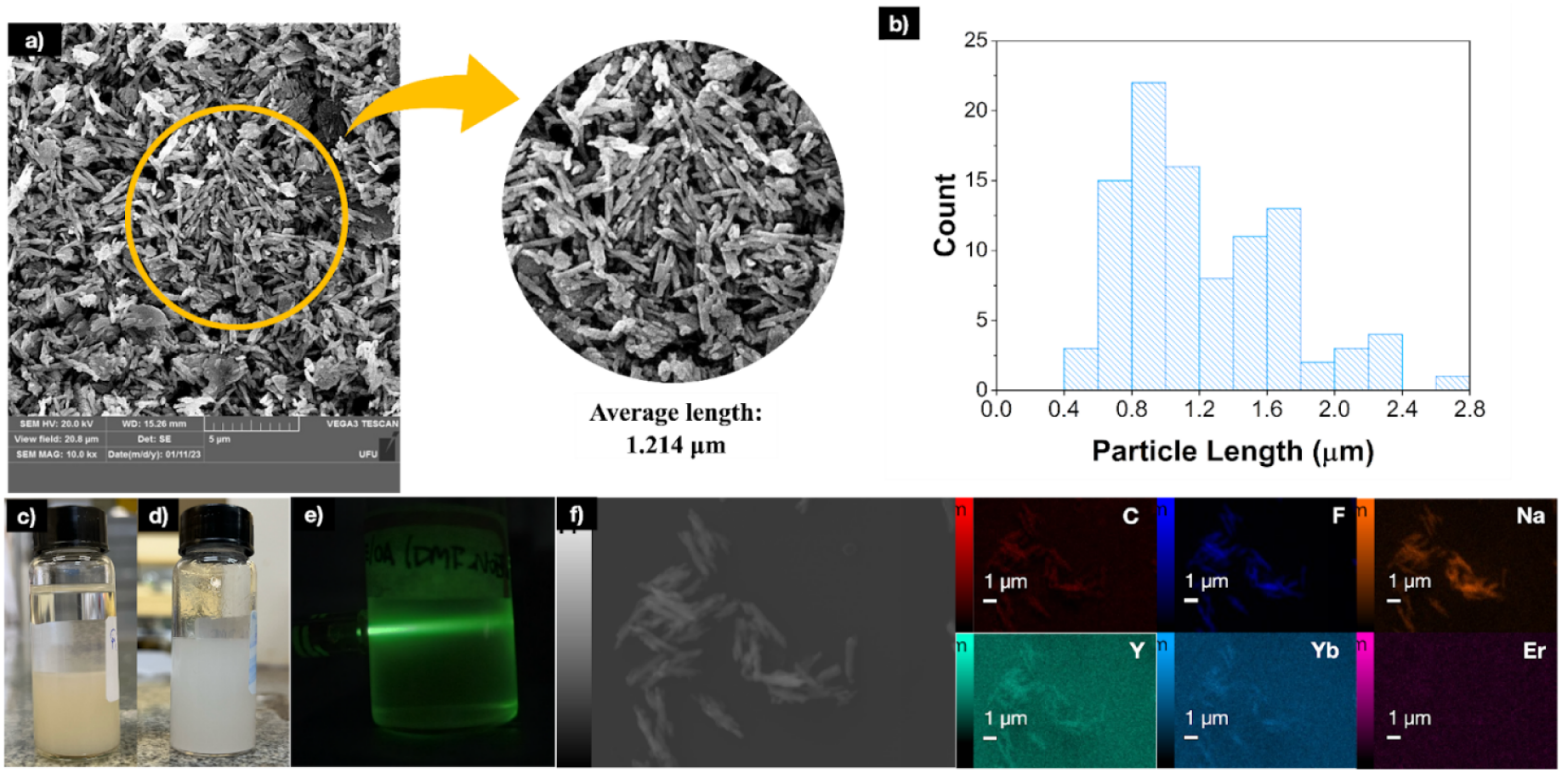
(a) SEM images at ×10,000 magnification showing the morphology of NaYF_4_:Yb^3+^, Er^3+^ UCPs; (b) UCP length histogram; (c) UCPs before surface modification with NOBF_4_; (d) UCPs after surface modification with NOBF_4_; (e) UCPs under excitation with a 980 nm laser; (f) UCPs EDX chemical map.

The NaYF4:Yb^3+^,Er^3+^ upconversion particles (UCPs) synthesized in this work via a hydrothermal route are depicted in the scanning electron microscopy (SEM) micrographs shown in Figure 1a. The images reveal needle-like microparticles with varying lengths, as illustrated in Figure 1b, which presents the particle size distribution histogram. The histogram indicates two predominant size ranges, centered at 1.0 and 1.8 μm, with an average particle length of 1.2 μm. At higher magnifications, overlapping particles are observed, appearing as small rods. The morphology of the synthesized microparticles is highly dependent on the experimental conditions employed during the synthesis, particularly the concentration of sodium hydroxide and, more broadly, the ratio between ethanol, oleic acid, and sodium hydroxide.^17^

Considering the characteristics of the upconversion nanoparticles (UCPs) produced in this study, particularly their hydrophobicity due to the oleic acid (OA) coating, dispersing them in polar solvents can lead to particle aggregation, which complicates the preparation of films and polymer membranes through solution casting. To prepare the cellulose acetate butyrate composite with UCPs, *N*,*N*-dimethylformamide (DMF) was used as the solvent for polymer dissolution and microparticle dispersion. A ligand exchange method was employed to enhance the interaction of the UCPs with the solvent and the polymer, replacing the OA with BF_4_^–^ ions.^18^

**Figure 2:**
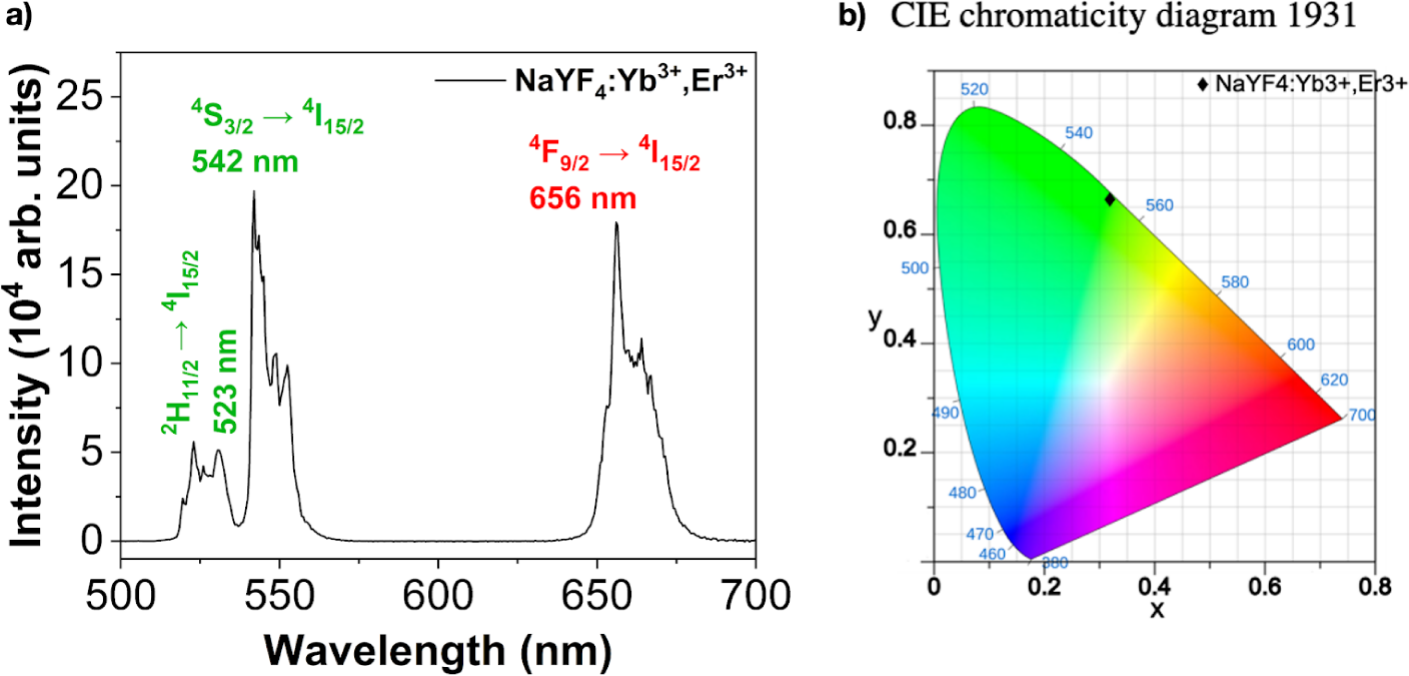
(a) NaYF_4_:Yb^3+^,Er^3+^ particles’ luminescence spectra; (b) chromaticity diagram of particles.

The dispersion of the modified microparticles was evaluated in cyclohexane and dimethylformamide, as shown in Figure 1c,d, respectively. It was observed that the modified microparticles do not disperse adequately in cyclohexane (Figure 1c), quickly settling at the bottom of the container (10 min), unlike what occurs with unmodified UCPs. In contrast, the test conducted with modified UCPs in DMF exhibited a more dispersed and uniform system, with the microparticles remaining suspended for 3 days. The colloidal dispersion of microparticles modified with NOBF_4_ in DMF showed good uniformity and stability due to surface modification by BF_4_^–^ ions. After excitation of the surface-modified microparticles dispersed in DMF using a 980 nm laser, green emission was observed with the naked eye (Figure 1e), demonstrating the energy upconversion process. This emission was further confirmed by the same upconversion luminescence spectrum under the same excitation conditions (Figure 2a). Figure 1f presents the chemical map of the microparticles, where it is evident that these particles contain chemical elements characteristic of NaYF_4_:Yb^3+^,Er^3+^ UCPs. This observation confirms the success of the synthesis process, which is further validated by the luminescence spectra and chromaticity diagram provided in Figure 2a,b.

**Figure 3:**
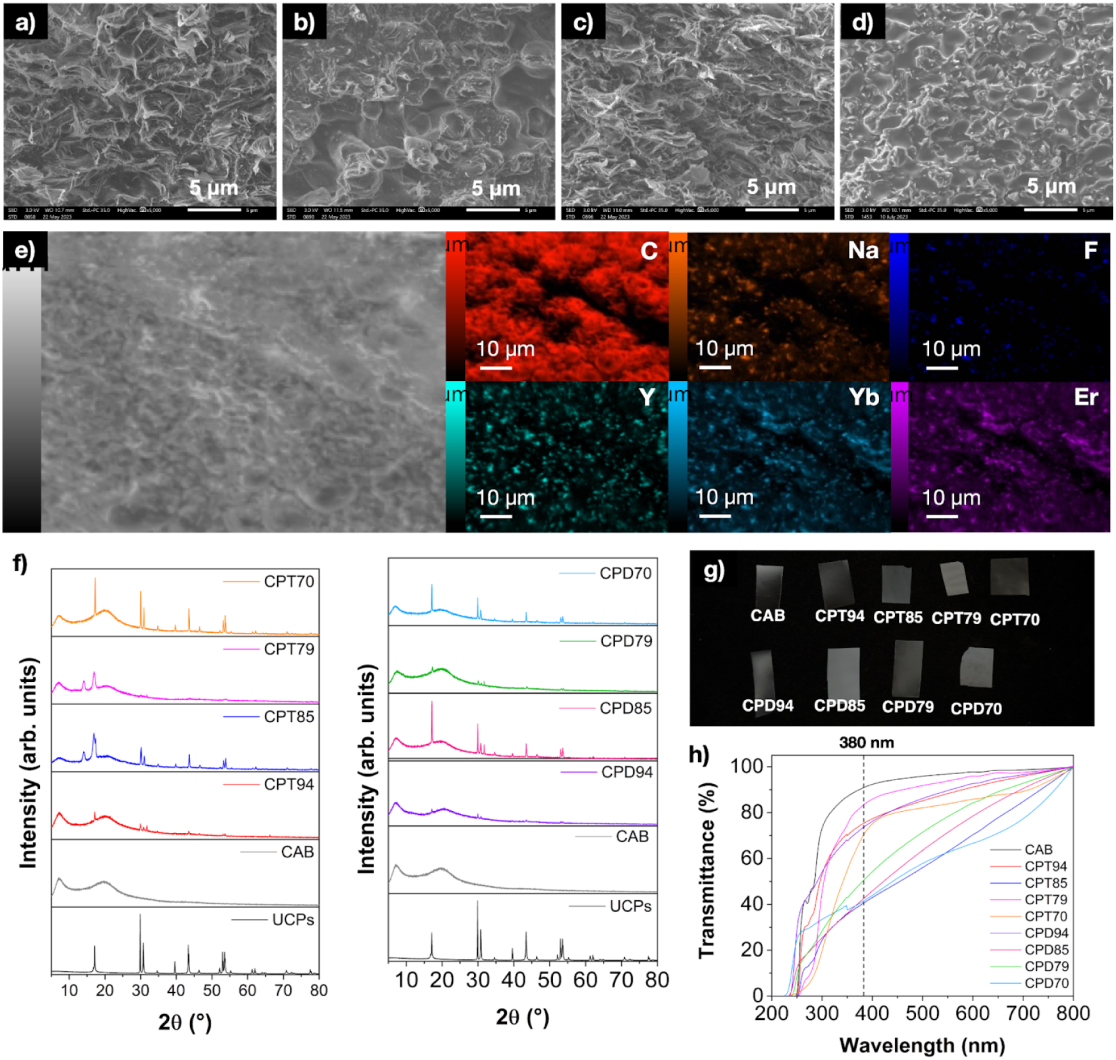
Fracture SEM micrographs of CAB/NaYF4:Yb^3+^,Er^3+^ composite films at 5,000× magnification: (a) CPT94, (b) CPT70, (c) CPD94 and (d) CPD70; (e) EDX chemical map for CPT85 film; (f) XRD diffractograms of UCPs and composite films; (g) CPT94 film and CPT70 film; (h) UV–vis transmittance spectra of films.

The morphology of the CAB films with UPCs prepared by casting solutions according to the procedure summarized in Table 1 can be observed in the scanning electron microscopy (SEM) micrographs presented in Figure 3a–e.

**Table 1:** TGA, DSC, and DMTA Data for Additives and CAB and Composite Films

		TGA data				DSC data			DMTA data (35 °C)
Sample	UCP/additive (w/w)	*T*_onset_ 1 (°C)	*T*_max_ (°C)	*T*_onset_ 2 (°C)	*T*_max_ 2 (°C)	*T*_melting_(°C)	Δ*H*_melting_ (J g^–1^)	*T*_g_ (°C)	*E*’ (MPa)
TWEEN 80	----------	223.6	272.0	298.3	360.2	----------	----------	----------	----------
DSS	----------	273.7	284.3	----------	----------	----------	----------	----------	----------
CAB	----------	312.0	366,6	----------	----------	167.41	18.070	131.90	2151.59
CPT94	1/5	338.9	362.2	----------	----------	166.98	15.717	94.91	2191.86
CPD94	1/5	261.3	268.3	315.0	339.5	168.14	14.598	96.52	1093.40
CPT85	10/5	332.7	358.0	----------	----------	167.01	16.091	84.84	2191.01
CPD85	10/5	244.7	257.6	319.4	338.8	166.93	13.597	94.98	2462.42
CPT79	1/20	333.1	363.5	----------	----------	164.80	5.328	95.04	1128.87
CPD79	1/20	254.0	259.8	319.4	331.6	166.23	14.922	101.43	2440.96
CPT70	10/20	324.4	357.4	----------	----------	165.72	8.317	85.32	24.6
CPD70	10/20	229.7	245.7	308.6	331.5	167.41	11.078	111.87	1571.16

The cross-sectional area of the CAB film without additives (image not shown) features smooth, layered structures, an aspect that was altered by the presence of UCPs dispersed in the polymer matrix. The composites exhibited heterogeneous structures organized in globular forms with dispersed microparticles. Significant morphological changes in the composite film images associated with the increased percentage of added microparticles and additives are shown in Figure 3. Images (a) and (c) depict the composites produced with 1% UCP microparticles, prepared with 5% Tween80 (CPT940) and 5% DSS (CPD94), respectively. Images (b) and (d) represent films with 10% microparticles and 20% additives (CPT70 and CPD70). In the latter images, the granular texture and presence of microparticles are clearly visible, attributed to the quantity of particles incorporated into the polymer matrix. These observations are further corroborated by Figure 3e, which displays the chemical mapping obtained through energy-dispersive X-ray spectroscopy (EDX). The EDX mapping reveals the distribution of the elements Na, F, Y, Yb, and Er, which constitute the structure of the NaYF4:Yb^3+^,Er^3+^ microparticles across the entire thickness of the film. This distribution demonstrates the effective dispersion achieved through surface modification with NOBF_4_.

The CAB (cellulose acetate butyrate) and its composites were characterized by using X-ray diffraction (XRD) to analyze the crystalline phase profile of both the polymer and the incorporated microparticles. The results are presented in Figure 3f. For the CAB matrix, two halos are observed: a low-intensity van der Waals halo at 2θ close to 7° and a more pronounced van der Waals halo with a maximum at 20°, which are characteristic of the predominantly amorphous nature of the polymer. The presence of the low van der Waals halo at 2θ below 10° is typical for cellulosic derivatives, such as cellulose acetate and cellulose acetate butyrate, and arises due to the bulky side groups (acetyl and butyryl) introduced during chemical modification, in contrast to unmodified cellulose. Additionally, the XRD data reveal a broadening of the crystalline peaks for the CPT85 and CPT79 composite samples, suggesting a reduction in crystallite size, likely due to the influence of the additives used during processing. It is worth noting that the polymer matrix retains a predominantly amorphous profile with low crystallinity, as further confirmed by the endothermic process observed in differential scanning calorimetry (DSC) analysis.

Furthermore, in the composite films, these peaks remain practically unchanged, indicating that the particles did not have a significant effect on the structure of the CAB matrix.^19^ Peaks related to the luminophores are also present, with the intensity increasing proportionally to the concentration of particles added during film preparation. The CPT94, CPD94, CPT79, and CPD79 samples, which contain 1% luminophores, exhibit an intensity lower than those of the films CPT85, CPD85, CPT70, and CPD70, which have 10% particles. The number of peaks and their positions conform to the reference standard for the hexagonal β-phase of NaYF4 (JCPDS File No. 00–064–0156), demonstrating the effectiveness of the synthesis procedure for these particles.

**Figure 4:**
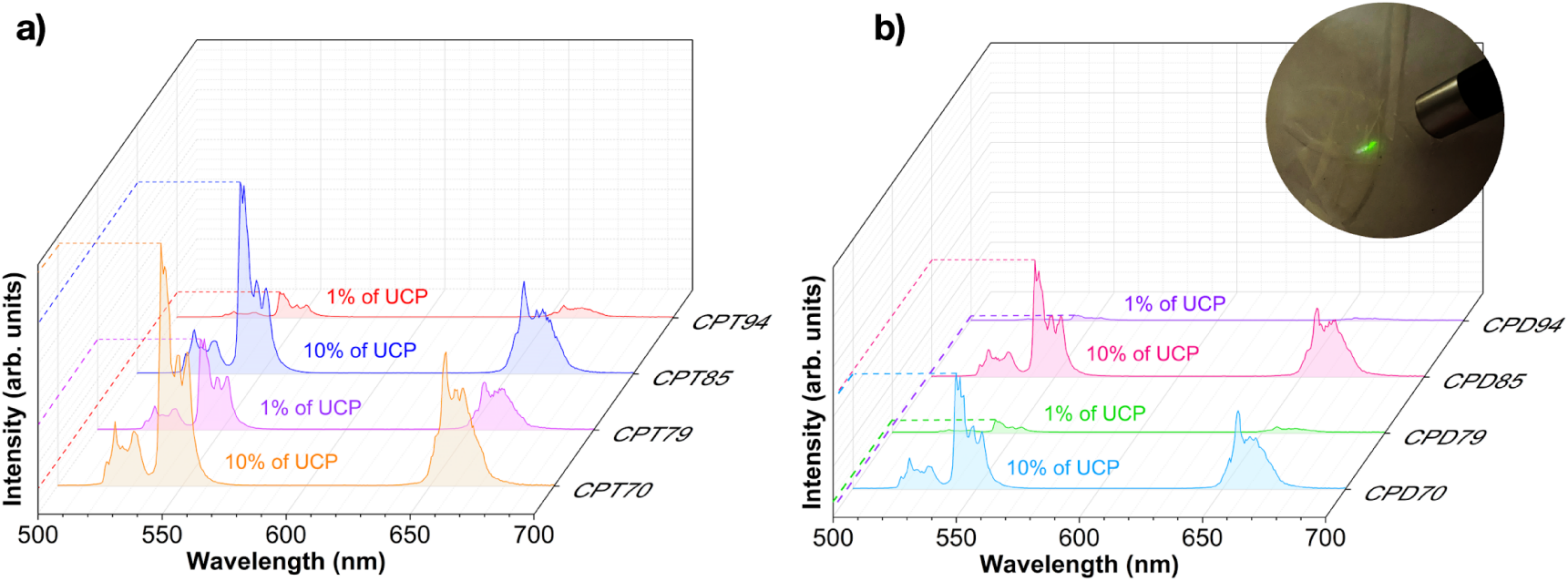
(a) CPT composite films’ luminescence spectra; (b) CPD composite films’ luminescence spectra and CPT94 film under 980 nm laser excitation.

The images of these films are shown in Figure 3g. The films exhibited moderate transparency, with some displaying a whitish appearance. The transparency of the CAB film and the CAB/NaYF_4_:Yb^3+^,Er^3+^ composites was measured by using UV–vis spectra, as shown in Figure 3h. The CAB film records a transparency of 90%, while the composites CPT94, CPT79, CPT70, and CPD94 exhibited transparency between 71% and 83% at the 380 nm wavelength. The other composite films showed transmittance values close to 40% and a whitish appearance, as shown in Figure 3g. The results emphasize the differences between the films produced with UCPs compatibilized with Tween 80, beyond the mere percentage of microparticles added. In the case of UCPs compatibilized with DSS, the CPD94 film displayed the highest transparency, with 83% transmittance; however, with the increase in microparticle content, the transmittance drastically decreases, indicating that for this surfactant agent, the best results are achieved with low concentrations of microparticles. However, it is essential to emphasize that there is no reduction in the luminescence capacity of the films with an increase in the amount of UCPs, as shown in Figure 4.

Figure 4a,b presents the luminescence spectra of the composite films, revealing that luminescence intensities increase with a higher percentage of UCP. It is also observed that films using DSS as an additive show a lower emission intensity than those using Tween 80. This reduction in intensity may be attributed to the type of additive and a possible suppression effect caused by the different functional groups in DSS on the luminophore, unlike what is seen with Tween 80. The image in Figure 4b shows the emission of intense green light when the composite film is excited by a 980 nm laser, demonstrating that the polymeric medium does not suppress the upconversion properties of the microparticles.

### 2.2 Thermal Analysis and Dynamic Mechanic Analysis

The observed effect on the optical properties of CAB/UCP composite films is a direct result of using compatibilizing agents, which play a critical role in the effective incorporation of upconversion particles (UCPs) into the polymer matrix. These agents significantly improve the dispersion of microparticles, prevent aggregation, and facilitate the formation of films with superior optical properties. The effectiveness of these additives can be further evaluated through thermal analysis, which provides insights into the thermal stability, crystallinity, and interfacial interactions within the composite.

**Figure 5:**
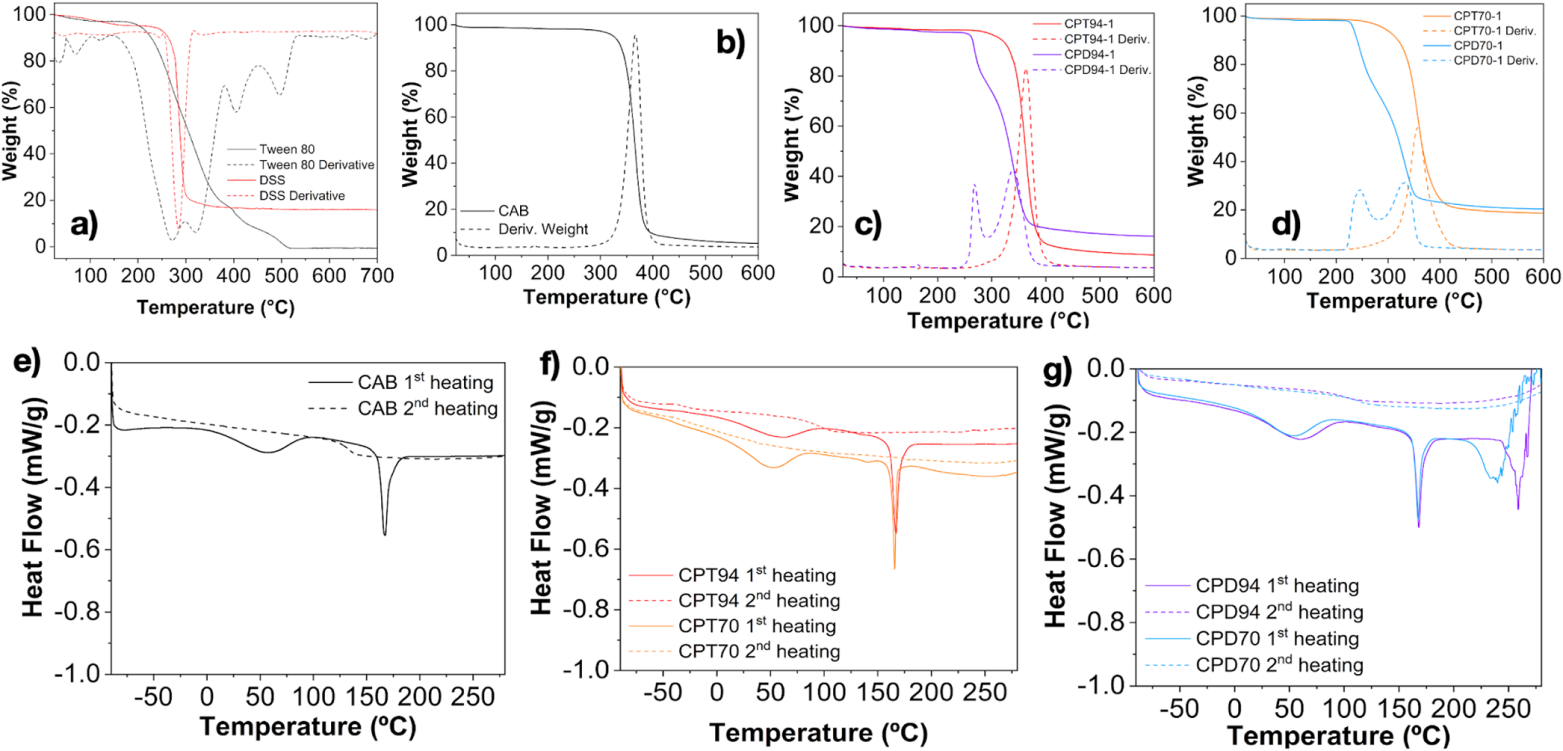
(a) Tween 80 and DSS; (b) CAB; (c) CPT94 and CPD94; and (d) CPT70 and CPD70 TGA thermograms. First and second DSC heating curves are presented for (e) neat CAB; (f) CPT94 and CPT70; and (g) CPD94 and CPD70.

Figure 5a,b presents the thermogravimetric (TG) curves of the additives (Tween 80 and DSS) and neat CAB film. Tween 80 exhibits four distinct thermal decomposition events, with the first two contributing significantly to mass loss. In contrast, DSS displays a single mass-loss event, indicating a simpler thermal degradation profile. The neat CAB polymer demonstrates a single prominent thermal event, with a maximum decomposition temperature of 366.6 °C. Table 1 summarizes the key thermal events observed for the additives, neat CAB films, and composite materials.

Understanding the individual thermogravimetric behavior of the polymer and additives is crucial, as it provides fundamental insights into their thermal stability and degradation mechanisms. Additionally, it helps to identify potential interactions between the polymer matrix and the additives, which can significantly influence the thermal properties of the composite. The thermal properties of the composite, when compared to those of its isolated components, provide valuable information about the quality of the association between the dispersed phase and the polymer matrix, mediated by the presence of the additives. These insights are essential for selecting the most suitable materials and optimizing the performance of the produced composites.

Figure 5c,d shows that films with microparticles and Tween 80 as an additive undergo only a single thermal event. Notably, even at a high concentration of 20%, the onset temperatures are higher than those observed for the pure polymer, and the maximum degradation temperature remains close to that of the neat CAB. This thermal behavior suggests that Tween 80 offers excellent compatibility with the polymer matrix, facilitating the effective incorporation of microparticles without compromising thermal stability. In contrast, films utilizing DSS as an additive display two distinct thermal events related to DSS, which occur at temperatures near those of CAB decomposition. This observation indicates a lower level of compatibility between DSS and the polymer matrix compared to that of Tween 80, as evidenced by the additional thermal events. These events suggest potential interactions or phase separations that could compromise the film’s structural integrity or uniformity. This comparison highlights the superiority of Tween 80 in creating a more unified and thermally stable composite film.

The DSC curves for CAB films and their composites, presented in Figure 5e–g, along with the data summarized in Table 1, reveal significant thermal characteristics of these materials. In the first heating scan of the CAB film, two distinct thermal events are observed. The first event, occurring between 20 and 80 °C, is attributed to the loss of adsorbed moisture on the surface. The second event, characterized by peaks between 164.80 and 168.14 °C, corresponds to the melting temperature (*T*_m_) of CAB. Additionally, the glass transition temperature (*T*_g_) is identified at 131.90 °C, providing further insight into the thermal behavior of the polymer matrix.

A reduction in the *T*_g_ value is noted in the composite films, which is more pronounced in films containing Tween 80. Small changes in the melting temperature and enthalpy are observed, suggesting effective interactions between the additives and the polymer matrix, particularly with Tween 80. This efficient interaction contributes to the good dispersion of the microparticles, resulting in improved material properties.

The presence of incorporated particles may also influence these interactions, as demonstrated by de Freitas Silva et al.^16^ in their study of CAB composite films containing [Eu(TTA)_3_] particles. Effective dispersion of the particles enhances the examined properties, ensuring that the mechanical performance of the composites is maintained or even superior to that of films without UCPs. This observation is supported by the results presented in Table 1, which show storage modulus values at 35 °C for both the CAB film and the composites. While some results are slightly lower than those of the pure film, most values are comparable to or exceed the reference, indicating that the microparticles are well-dispersed and stabilized within the produced films.

In this work, it is demonstrated that the use of cellulose acetate butyrate (CAB) as a polymer matrix for NaYF4:Yb^3+^,Er^3+^ particles with upconversion energy properties yields stable composites, particularly when the percentage of microparticles and the appropriate use of additives are optimized. Table 2 provides a qualitative comparison of this work to previously developed studies, showing that this system is well-suited for producing composites with promising properties for various applications. The results highlight the good performance of the CAB-based composites in terms of stability, transparency, and mechanical properties, as demonstrated in this study.

**Table 2:** Polymeric Matrices Used in the Development of Composites with NaYF4:Yb^3+^,Er^3+^ Nano/Microparticles

Polymeric Matrix	PVA^8^	PA6/PMMA^11^	PMMA^20^	CNC^13^	CA^15^	CAB^16^	CAB (this work)
UC particles	NaYF4:Yb^3+^,Er^3+^	NaYF4:Yb^3+^,Er^3+^	NaYF4:Yb^3+^,Er^3+^ (Tm^3+^)	β-NaYF_4_:Yb^3+^,Tm^3+^	NaYF4:Yb^3+^,Er^3+^,Ce^3+^	Eu(TTA)_3_	NaYF4:Yb^3+^,Er^3+^
Main objective	Synthesize luminescent PVA films with UCNPs for anticounterfeiting applications.	Fabricate transparent, upconversion photoluminescent nanofiber mats with tunable optical properties	Synthesize transparent upconversion nanocomposites via in situ photopolymerization	Develop a sensitive and reusable lycopene sensor using upconversion paper.	Theranostic platform for diagnosis and drug delivery consisting of UCNPs encapsulated with cellulose acetate (CA)	Develop optical thermometry using Eu(TTA)_3_ in a CAB matrix	Develop CAB-based upconversion composite films; optimize their structural, thermal, and luminescent properties
Particle Synthesis	Hydrothermal	Solvothermal, ligand exchange	Hydrothermal	Thermal decomposition	Hydrothermal method	Europium complex synthesized using thenoyltrifluoroacetone (HTTA) ligand.	Hydrothermal
Particle size	UCNPs: Not specified. Composite film thickness: ∼100 μm	UCNPs: 10–11 nm (average diameter); nanofibers: 100–250 nm	NaYF_4_ nanoparticles: ∼40 nm	UCNPs: ∼25 nm (average diameter)	CA capsules 320 ± 5 nm UCNPs spherical shape diameter of 50 ± 2 nm and rod shape width of 25 ± 1 nm, and the length of 80 ± 5 nm	Not applicable (europium complex, not UCNPs)	Microparticles average length of 1.2 μm.
Film preparation or capsule formation	Solution casting	Co-electrospinning Method and the Spin-Coating Process.	Bulk nanocomposites, in situ Photopolymerization	Solution casting of CNC/PVA mixture, then incorporating UCNPs via casting; spin-coating with PMMA.	UCNP-CA capsules were prepared by a solvent evaporation technique, allowing control of particle size by varying stirring rate.	Solution casting	Solution Casting
Transmittance (%)	74–93% in visible range, decreasing with UCNP content	79–86% transmittance in the visible range, decreases with UCNP content	55–90% (depending on UCP concentration)	High transparency (98%) reported for optimized sensor paper.	--------------------	Transparence decreases slightly with increasing europium concentration	Hight to Moderate transparency; higher transparency with DSS than Tween 80
Luminescence Intensity	Upconversion; strong emission; intensity increases with UCNP concentration.	Increases with UCNP concentration	Upconversion (green and blue); intensity increases with NaYF_4_ Tm^3+^ concentration	Upconversion; strong emission at 475 nm; intensity decreases with increasing lycopene concentration.	Efficient luminescence properties of UCNPs. 30% reduction of luminescence CA UCNP encapsulated	Downconversion; europium complex exhibits red emission; intensity decreases exponentially with temperature	Upconversion; intensity higher with Tween 80 than DSS; intensity increases with UCP concentration.

It is important to emphasize that the use of CAB with the NaYF4:Yb^3+^,Er^3+^ system has not been reported in the literature to date. The only related work cited in Table 2 employs a different system, Eu(TTA)_3_, further underscoring the novelty and significance of this study. This unique combination of CAB and NaYF4:Yb^3+^,Er^3+^ microparticles opens new possibilities for advanced applications in optical devices, sensors, and other functional materials.

In summary, the DSC curves and thermal data analysis confirm the effective compatibilization between the CAB polymer matrix and the UCPs, facilitated by the addition of additives such as Tween 80 and DSS. This suitable alignment promotes efficient dispersion of the microparticles and enhances the composites’ thermal and mechanical properties. This compatibilization is crucial for optimizing the efficiency of the material’s upconversion properties.

As shown in Table 3, the main results highlight the production of materials with distinct properties, particularly the samples CPT94, CPD94, CPT79, and CPT70, which exhibit good transmittance, luminescence intensity, and thermal stability, representing an excellent balance among all of the studied properties. The mechanical properties, however, require further optimization, which can be achieved by controlling the proportion of particles and additives.

**Table 3:** Evaluation of the Main Properties Analyzed for CAB/NaYF4:Yb^3+^,Er^3+^ Composites, Including Transmittance, Luminescence Intensity, Thermal Stability, and Mechanical Performance

			Luminescence *I*/10^3^AU			TGA		DSC			DMTA
Sample	UCP/additive (w/w)	UV/vis T (%)	523 nm	542 nm	654 nm	*T*_Onset_ (°C)	*T*_dmax_ (°C)	*T*_m_ (°C)	Δ*H*_m_ (J g^–1^)	*T*_g_ (°C)	*E*’ (MPa)
CAB	----------	90.6	---------	---------	---------	312.0	366,6	167.41	18.070	131.90	2151.59
CPT94	1/5	75.0	13.0	55.8	11.5	338.9	362.2	166.98	15.717	94.91	2191.86
CPD94	1/5	73.2	2.7	12.4	2.2	261.3	268.3	168.14	14.598	96.52	1093.40
CPT85	10/5	40.2	104.8	450.9	112.6	332.7	358.0	167.01	16.091	84.84	2191.01
CPD85	10/5	42.0	64.0	278.4	67.0	244.7	257.6	166.93	13.597	94.98	2462.42
CPT79	1/20	82.8	55.8	213.2	57.4	333.1	363.5	164.80	5.328	95.04	1128.87
CPD79	1/20	48.9	5.9	29.0	5.4	254.0	259.8	166.23	14.922	101.43	2440.96
CPT70	10/20	70.8	153.0	540.3	144.0	324.4	357.4	165.72	8.317	85.32	24.6
CPD70	10/20	41,1	73,5	276,2	78.7	229.7	245.7	167.41	11.078	111.87	1571.16

These composites emerge as promising candidates for applications in devices that utilize energy upconversion such as solar cells, optical sensors, and photonic devices. The ability to maintain or even improve mechanical, thermal, and optical properties makes these composites ideal for technological innovations in demanding sectors.

## 3 Conclusions

This work successfully demonstrated the fabrication of cellulose acetate butyrate (CAB) films as a highly effective platform for developing nanocomposites with NaYF_4_:Yb^3+^,Er^3+^ upconversion particles (UCPs). The synthesis and surface modification of UCPs greatly enhanced their dispersion within the polar solvent and polymer matrix, forming homogeneously distributed composite films. Additives such as Tween 80 and dioctyl sodium sulfosuccinate (DSS) significantly influenced the mechanical and optical properties of the films (e.g., CPT94, CPD94, and CPT79), with Tween 80 leading to composites with superior luminescence performance (especially CPT94, CPD94, CPT79, and CPT70). Thermal analyses confirmed the compatibility between CAB and these additives, highlighting Tween 80 as a more effective plasticizer for CAB than DSS. The upconversion luminescence properties of the films were well-retained, confirming CAB as a suitable protective matrix that preserves the optical characteristics of the UCPs. This study opens new possibilities for applying CAB/UCP composites in energy conversion, sensing, and photonic devices. Future research should focus on optimizing particle loading and exploring additional functional additives to further enhance the performance of these composites for specific applications.

## 4 Experimental Section

### 4.1 Materials

Hexahydrate erbium chloride (ErCl_3_·6H_2_O), hexahydrate ytterbium chloride (YbCl_3_·6H_2_O), hexahydrate yttrium chloride (YCl_3_·6H_2_O), nitrosonium tetrafluoroborate (NOBF_4_), polyoxyethylene sorbitan monooleate (Tween 80), dioctyl sulfosuccinate sodium (DSS), and cellulose acetate butyrate (CAB) with M.W. = 70,000 were purchased from Sigma-Aldrich. Oleic acid (OA) was purchased from Dinâmica Qumica Contemporânea Ltda. Chloroform (CHCl_3_) was purchased from Synth. Ethanol 95% PA ACS was purchased from Neon Comercial Reagentes Analíticos Ltda. *N*,*N*-Dimethylformamide was purchased from Êxodo Científica. Sodium hydroxide (NaOH) was purchased from Vetec Química Fina.

### 4.2 Upconvertion Particle (UCP) Synthesis

The synthesis process was based on procedures described by Wu et al.^21^ and Van Duong et al.^22^ An aqueous solution (deionized water) of 0.25 mol L^–1^ of rare earth chlorides (LnCl_3_·6H_2_O) was prepared in a Y/Yb/Er (yttrium/ytterbium/erbium) proportion of 78/20/2 (mol/mol). In a beaker containing 26 mmol of oleic acid (OA), 9 mmol of sodium hydroxide (NaOH), 10.4 mL of ethanol, and 1.6 mL of deionized water, 4 mL of rare earth chloride solutions were added. Simultaneously, in a second beaker, 44 mmol of sodium fluoride (NaF), 5 mL of deionized water, and 5 mL of ethanol were combined. Both beakers were kept under constant stirring for 30 min, after which their contents were mixed and stirred for an additional hour. Subsequently, the resulting mixture was transferred to a 316-steel reactor lined with a Teflon inner jacket and kept under heating in an oil bath at a temperature of 190 °C for 24 h. The reactor was cooled to room temperature, and the particles were recovered by centrifugation with excess ethanol. Several washes were carried out with ethanol and cyclohexane, and finally, the NaYF_4_:Yb^3+^,Er^3+^ particles were dispersed in cyclohexane.

### 4.3 Colloidal Dispersion Stabilization

The particle stabilization mechanism was based on the process described by Serge-Correales et al.^18^ with modifications. In a tube, 4 mL of NaYF_4_:Yb^3+^,Er^3+^ dispersion in cyclohexane, 1 mL of cyclohexane, 5 mL of *N*,*N*-dimethylformamide (DMF), and 120 mg of nitrosonium tetrafluoroborate (NOBF_4_) were added. The mixture was stirred vigorously and left to rest until it was separated into two phases. The upper phase was discarded, and the lower phase was transferred to a 50 mL centrifuge tube with excess chloroform. The tube contents were centrifuged at 3622 g of RCF and then washed repeatedly with 1 mL of DMF and 8 mL of chloroform. Finally, the modified particles were dispersed in DMF.

### 4.4 Production of Composite Films

The composite films were produced using the casting method from a solution made by dispersing 1 g of material composed of cellulose acetate butyrate (CAB), Tween 80 or dioctyl sodium sulfosuccinate (DSS), and upconversion microparticles in 30 mL of DMF. The proportions of polymer, additives, and upconversion microparticles, along with the film identifications, are detailed in Table 4. The mixture was stirred at 70 °C until complete dissolution and poured into a glass Petri dish. Subsequently, the dish was placed in an air circulation oven to dry at 50 °C for 24 h.

**Table 4:** Proportion between CAB, Microparticles, NaYF_4_:Yb^3+^,Er^3^, and Additives for Composite Production

Composite Film	CAB (%w/w)	NaYF_4_:Yb^3+^,Er^3+^ (%w/w)	Tween 80 (%w/w)	DSS (% m/m)
CPT94	94	1	5	-----
CPT85	85	10	5	-----
CPT79	79	1	20	-----
CPT70	70	10	20	-----
CPD94	94	1	-----	5
CPD85	85	10	-----	5
CPD79	79	1	-----	20
CPD70	70	10	-----	20

### 4.5 Particle and Composite Film Characterization

X-ray diffraction of particles and composite films was performed using a Rigaku Smartlab SE X-ray diffractometer operating at 40 kV with a 20 mA current and a CuKα cell (1.54186 Å). The samples were analyzed in a glass sample holder with a 2θ scanning range of 5 to 80°, a scanning speed of 5° min^–1^, and resolution of 0.02°.

A TESCAN VEGA3 scanning electron microscope was used to observe the morphologies of the microparticles. The samples were coated with gold, and the magnifications analyzed were 1,000–10,000× with an HV of 20 kV. SEM images of the composite films were obtained with a JEOL JSM-IT500HR high-resolution scanning electron microscope (SEM-FEG). The samples were covered with carbon and analyzed at magnifications of 1,000× to 20,000× with an HV of 3 kV. The film microscopies were performed in the fracture session. The average length of the luminescent particles was calculated using ImageJ software. For the chemical analysis of EDX, an XEDS accessory coupled to the SEM-FEG was used with an HV of 20 kV. Thermal analysis of the samples was conducted in two stages: thermogravimetry (TGA) and differential scanning calorimetry (DSC). The TGA analysis of the film samples was carried out in a TA Instruments SDT Q600 thermogravimetric oven under a N_2_ atmosphere, with a temperature range of 25 to 600 °C and a heating rate of 10 °C min^–1^. The DSC was carried out on equipment model D25, DISCOVERY series from TA Instruments, in the range of −100 to 280 °C with a N_2_ flow and heating rate of 10 °C min^–1^. Photoluminescence spectroscopy of the particles and composite films was performed on a HORIBA Jobin Yvon Fluorolog-3 FL3–122 spectrofluorometer coupled to a 980 nm Yb-doped optical fiber laser as an energetic excitation source. The dynamic mechanical thermal analysis (DMTA) was performed in TA Instrument Q800 equipment. The analysis conditions were based on ASTM D4065:2020, heating the samples from 23 to 153 °C, with a heating rate of 2 °C min^–1^, a frequency of 1 Hz, a deformation amplitude of 10 μm, and a preload of 0,1 N.
